# A novel bias-free approach for robust perceptual threshold estimation

**DOI:** 10.3758/s13428-026-03038-5

**Published:** 2026-05-05

**Authors:** Luca Tarasi, Margherita Covelli, Caterina Bertini, Vincenzo Romei

**Affiliations:** 1 Centro Studi e Ricerche in Neuroscienze Cognitive, Dipartimento di Psicologia, Alma Mater Studiorum, Università di Bologna, Campus di Cesena, viale Rasi e Spinelli, 176, Cesena, 47521 Italy; 2 Universidad Antonio de Nebrija, Madrid, Spain

**Keywords:** Perceptual threshold, Decisional bias, Signal detection theory, Threshold estimation methods, Bias-free approach

## Abstract

Perceptual threshold estimation stands as a fundamental variable manipulation in psychology and neuroscience, fine-tuning sensory input intensity to align perceptual precision across individuals. Current methods, such as constant stimuli and staircase procedures, inaccurately assume that perceptual thresholds are influenced solely by sensitivity, overlooking the role of response biases and consequently leading to unreliable estimates. The underlying reason is that classical methods traditionally focus on hit rates—the correct detection of stimuli—while neglecting false alarms, where participants incorrectly report a stimulus when none is present. According to signal detection theory, hit rates can arise from genuine sensitivity or a liberal response criterion, making it crucial to account for both measures. To overcome this confound, we developed bias-free versions of these methods by including target-absent trials to factor in decisional bias during threshold evaluation. Across 74 participants, we robustly demonstrated that widely used classical procedures overestimate perceptual thresholds due to uncontrolled interindividual variability in participant criterion. Conversely, the bias-free staircase procedure achieved reliable sensitivity thresholds, effectively mitigating the influence of decisional bias. We strongly advocate for the adoption of this straightforward, bias-free approach. Its effectiveness, simplicity, and quick execution make it highly feasible for widespread use in enhancing the reliability of threshold estimation and reducing variability in future scientific investigations across various fields.

**Supplementary Information:**

The online version contains supplementary material available at 10.3758/s13428-026-03038-5.

## Introduction

Perceptual threshold refers to the minimum intensity of a stimulus needed for our senses to correctly detect it in a set proportion of trials. Over time, various psychophysical methods and experimental techniques have been developed to quantify the relationship between the physical intensity of specific stimuli (light intensity) and the subsequent psychological response (brightness perception). These approaches find application across a spectrum of contexts, from psychology to physiology and neuroscience. Moreover, threshold estimation procedures often serve as an initial step for exploring how experimental manipulations might influence behavioral performance (Benwell et al., 2017; Chiappini et al., 2022; Cruz et al., 2025; Di Gregorio et al., 2022; Gallina et al., 2024; Powers et al., 2017; Romei et al., 2013; Samaha et al., 2017; Tarasi, Bertaccini, et al., 2025; Tarasi, Covelli, et al., 2025). Consequently, inaccuracies in threshold measurement could lead to a cascade effect, compromising the reliability of subsequent measurements, results, and interpretations. Here, we assert that classical methods widely used in the current literature are marked by a fundamental flaw: they consistently overlook the influence of decisional bias during threshold estimation. This issue appears evident when examining the two primary methods employed for calculating perceptual thresholds: the constant stimuli method (Guilford, 1954) and the staircase method (Breakwell, 1995). In the constant stimuli method, a range of stimulus intensities is pre-selected, ensuring that the lowest-intensity stimulus elicits perception in 0–5% of cases, while the highest intensity is perceived in 95–100% of cases (Burro et al., 2011). The selected intensity levels of the stimulus are randomized, with the hit rate (HR) percentages recorded and typically represented by a sigmoid curve. Conversely, the staircase method involves multiple ascending and descending series of stimuli, with their intensity determined by participants' previous response. Crucially, both methods consider only the hits (i.e., correct reports of the target), overlooking scenarios where participants may make a false alarm (i.e., incorrect reports of the target). However, according to signal detection theory (SDT; Green & Swets, 1966), a higher HR could stem from both higher perceptual sensitivity (i.e., heightened ability to perceive the target) and the adoption of a liberal response criterion (i.e., higher proclivity in reporting the target). Importantly, traditional threshold estimation methods disregard the impact of the decisional criterion because they do not investigate the false alarm rate (FAR), making it impossible to distinguish whether a participant is genuinely proficient in reporting the target or if the reports are inflated due to liberal criteria. Perhaps, more importantly, it does not allow one to control for the level of decisional bias across participants.

To account for and control the crucial role of decisional bias, we devised an SDT approach to perceptual threshold measurement by incorporating target-absent trials into the estimate. By including these instances, threshold estimation is no longer influenced by the decisional criterion, as it is possible to consider the FAR, and thus compute bias-controlled sensitivity thresholds. As a result, perceptual thresholds for individuals with a more liberal versus conservative criterion should no longer differ in terms of sensitivity, as the higher versus lower number of hits they achieve (potentially leading to substantially different threshold estimates with classical methods) will be counterbalanced by a corresponding higher versus lower number of FAs. To test this hypothesis on empirical grounds, we have devised an alternative iteration of both the staircase method (*bias-free staircase method*) and the constant stimuli method (*bias-free constant stimuli method*). This allowed us to explore the impact of the decisional criterion on both adaptive and nonadaptive traditional approaches. To validate these new procedures, we opted for a threshold indicator of 70% detection accuracy and compared the performance of the two classical methods with that of the two adapted versions. In addition, through mathematical simulations, we estimated that 70% accuracy would correspond to sensitivity of approximately 1.46. Therefore, we also evaluated which method more closely approached the target sensitivity value. Our main research hypothesis posits that the bias-free methods will enable us to acquire perceptual threshold values that are more accurate and stable than those obtained using classical methods.

## Methods

### Participants

For our study, we recruited participants aged 18 to 35 years, as this age range allowed us to capture a representative sample of the population while minimizing the impact of age-related variables that might otherwise influence our results. Additionally, this age range is commonly used in psychological research, making our study consistent with existing literature. We employed convenience sampling, selecting participants based on their availability and willingness to take part. This approach enabled us to efficiently gather data within the targeted age range. Given the nature of the study as a validation work for a new method that has never been used before, which could have potential applications across various future research areas, we opted for a robust approach by testing the procedure on a substantial number of participants. A total of 74 adult participants aged between 21 and 34 years were tested. Five participants were removed from the analysis because they exhibited average accuracy in the test block exceeding 95% (four participants) or falling below 50% (one participant) across the four methods employed, leaving a final sample of 69 participants (male = 27, female = 42). In conducting this research, all relevant ethical guidelines were strictly followed. Informed consent was obtained from all participants before participation, and all data were collected and handled confidentially. No identifiable personal information is disclosed in this study.

### Experimental procedures

**Fig. 1:**
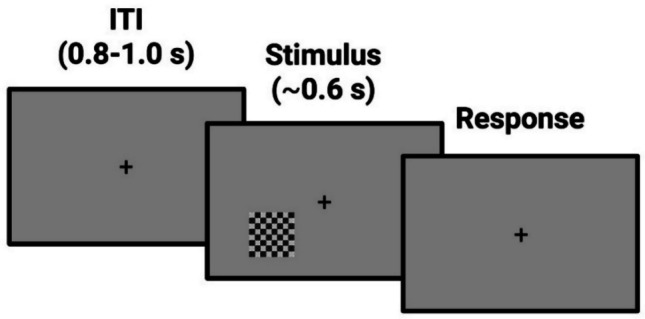
Experimental design. Behavioral data were collected during a simple visual detection task. Each trial started with a fixation cross positioned in the middle of the screen, followed by the appearance of a checkerboard, always positioned on the left lower part of the screen, either containing or not containing gray circles at different possible contrast levels. Following stimulus presentation, participants had to respond by pressing different buttons on the keyboard for perceived target presence or absence

Participants were invited into a dimly lit room and asked to sit on a chair positioned 57 cm from an LCD monitor (display resolution of 1280 × 1024 pixels, refresh rate 60 Hz). Stimuli were created and presented using MATLAB (version 2022, The MathWorks Inc., Natick, MA, USA) and Psychophysics Toolbox (Brainard, 1997), and consisted of checkerboards composed of alternating black and white squares. Each square could either contain gray circles (targets) or not (catch trials), with varying contrast intensities, creating more or less challenging contrasts to detect (Fig. 1). Stimuli had spatial frequency of 5.16 cycles/degree and were presented only in the lower left part of the screen at 4.1°/3.7° eccentricity (horizontal/vertical). The luminance of the monitor was gamma-corrected.

**Fig. 2:**
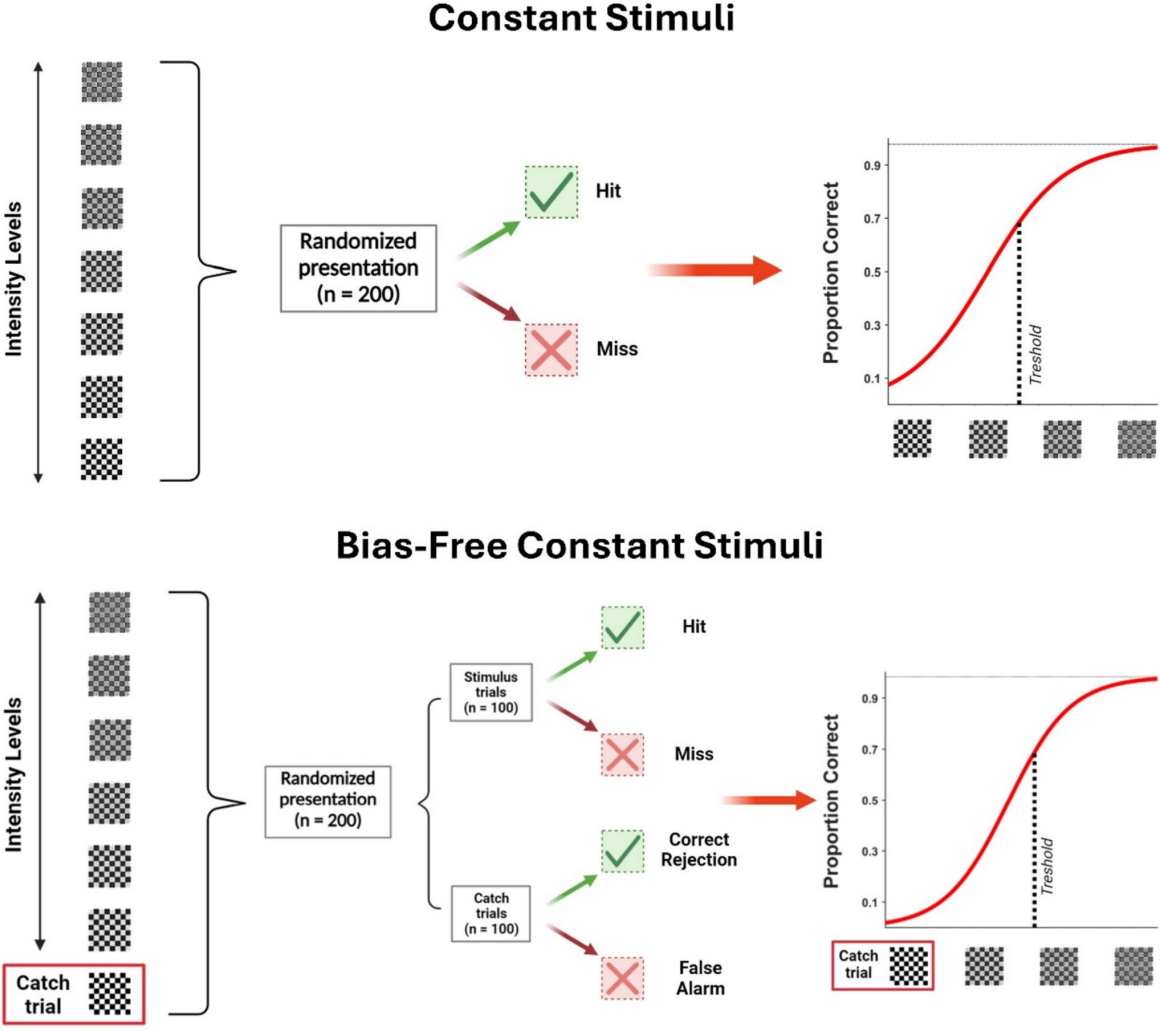
Constant stimuli methods. Graphical representation of the classic constant stimuli method (top) and its adapted bias-free version (bottom). The classic constant stimuli method involved presenting 200 randomized trials featuring one of seven different stimulus intensity levels. The lowest intensity (15/240 RGB value) aimed for nearly 0% correct responses, while the highest intensity (75/180 RGB value) was expected to yield a percentage of correct responses approaching 100%. In the adapted bias-free constant stimuli method, the first intensity level was replaced with catch trials (0/255 RGB value). Apart from this change, the method used the same other six stimulus intensities as the classic version, presenting them in 100 randomized trials, along with an additional 100 catch trials. On the right side of both graphs, the sigmoidal curves derived from fitting the data for each method are presented

**Fig. 3:**
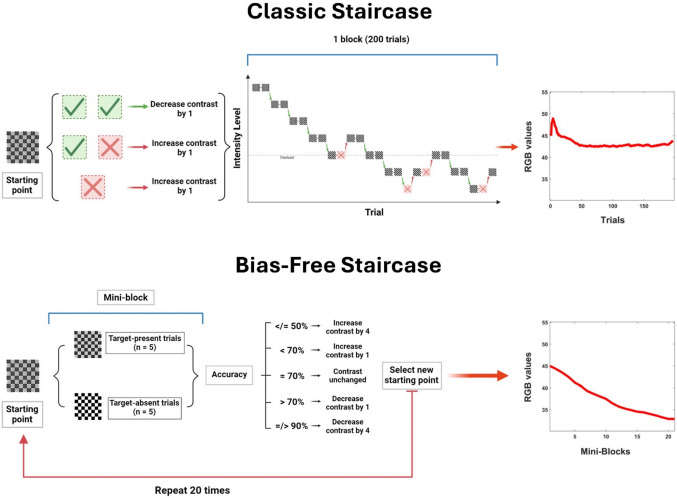
Staircase methods. Graphical representation of the classic staircase method (top) and its adapted bias-free version (bottom). The classic staircase method employs an adaptive algorithm of 200 trials that adjusts the contrast of stimuli based on responses to previous trials. Using a 2-down/1-up rule, the contrast was reduced after two consecutive correct detections of the stimulus. Conversely, the contrast was increased after a miss, regardless of whether the miss happened in the first or second presentation of the same target. The participant's threshold was calculated by averaging the contrast levels from the last three trials. The bias-free staircase method involved the administration of 20 mini-blocks, each containing five target-present and five target-absent trials in randomized order. After each mini-block, the accuracy reached by the participant was assessed to adjust the target contrast for the following mini-block. When the participant's accuracy was 70%, the contrast remained unchanged. If the accuracy was 50% or lower, the contrast increased by 4 RGB points. For accuracy under 70%, the contrast increased by just 1 RGB point. On the other hand, if accuracy was above 70%, the contrast was decreased by 1 RGB point. If it was above 90%, it was decreased by 4 RGB points. The participant's threshold was calculated by averaging the contrast levels from the last three mini-blocks. On the right side of each graph, the data fitting for each method, based on the collected data, is displayed

Participants were instructed to fixate on a central cross on the screen and indicate, as quickly and accurately as possible, the presence or absence of gray circles. Stimuli appeared for 50 ms, and participants were asked to press the *K* button if they perceived the stimulus (i.e., gray circles) and the *M* button if they did not. The task was preceded by a demo, during which stimuli were presented for a longer period on the screen, and a training part to familiarize participants with the task. The actual experiment was divided into two parts, each consisting of four blocks testing four different methods for perceptual threshold estimation, presented in random order across participants. The four methods employed included traditional up/down staircase (Breakwell, 1995) and constant stimuli methods (Bock & Jones, 1968; Guilford, 1954), as well as their modified versions incorporating catch trials (50%) to keep the spurious effects due to the decision criterion during threshold estimation under control. In the first part, corresponding to the threshold estimation, each block (200 trials) implemented a specific method for detecting the perceptual threshold (see Figs. 2 and 3). The target threshold was the contrast level at which the participant responded correctly in 70% of cases (i.e., based on hit rate and accuracy index, see below). The 70% target represents a well-established psychophysical standard (e.g., the classic 2-down/1-up staircase converges to approximately 70.7% by design (Levitt, 1971) and corresponds to an optimal range that avoids both floor effects (≤ 50% = chance) and ceiling effects (≥ 90% = insensitivity to individual differences). In the second part of the study, corresponding to the test detection task, four blocks of simple visual detection (100 trials each) were presented, in which 50% contained the targets while 50% contained catch trials (empty checkerboards). These test blocks were included to determine the actual percentage of correct responses obtained from the participants with each different estimated threshold. The inclusion of catch trials was essential for calculating the false alarm rate, which, in turn, allowed for the extraction of sensitivity and criterion indices from the SDT.

In both constant stimuli methods, the stimuli contrast to be presented was chosen in advance. In its classical formulation (*constant stimuli*), stimuli ranged from a contrast level of 15/240 RGB value (at which participants tend to give a percentage of correct responses approaching 0%) to 75/180 RGB (at which participants tend to give a percentage of correct responses approaching 100%). Seven different contrast levels were presented to ensure a proper sigmoidal fit to the data. In the constant stimuli method with the addition of catch trials (*bias-free constant stimuli*), these trials, where only noise was present (empty checkerboard), constituted 50% of the trials (i.e., 100 trials) and were used in place of the 15/240 RGB contrast stimulus. Stimuli at variable contrasts (the same ones used in the classical formulation) were presented in the remaining half. We chose to present 100 catch trials to match the number of trials we had in the bias-free staircase condition (see below). In both constant stimuli methods, the threshold was measured by calculating the contrast value corresponding to a percentage of correct responses equal to 70% as estimated using the *psignifit* package (Frund et al., 2011).

In the classic staircase method, an adaptive algorithm was used that varied the presented contrast based on the participant response in the previous trial. The rule used to modulate the contrast level in the next trial was the 2-down/1-up rule, which would result in 70% correct identification according to previous literature (Leek, 2001). Specifically, the contrast value was reduced after every two correct responses (correct detection of the stimulus) and increased by one level for each error made (miss of the target). In the bias-free staircase method, trials were divided into mini-blocks of 10 trials each: in five trials, the stimulus was present (*signal trial*), and in the other five (*catch trials*), only noise was present (empty checkerboard). The contrast level presented in each block was determined by the participant's accuracy in the previous block. Specifically, we summed the hit rate (hits divided by the number of signal trials) and the correct-rejection rate (correct rejections divided by the number of noise trials) and divided by 2 to derive an accuracy index. The rules for modulating the threshold of the next mini-block based on the accuracy obtained in the previous one were as follows:

- If accuracy was at or above 90%, the contrast level was reduced by 4 RGB points.
- If accuracy was above 70% and below 90%, the contrast level was reduced by 1 RGB point.
- If accuracy was equal to 70%, the contrast level remained unchanged.
- If accuracy was  below 70%  and above 50%, the contrast level was increased by 1 RGB point.
- If accuracy was at or below 50%, the contrast level was increased by 4 RGB points.

In both staircase methods, the starting contrast was set at a 45/210 RGB value, as it represented a stimulus midway between 15/240 (i.e., lower-contrast presentable stimulus) and 75/180 (i.e., higher-contrast presentable stimulus) RGB values. The threshold level was computed by averaging the values derived from the last three trials for the classic staircase methods and from the last three blocks for the bias-free staircase method.

### Estimation of expected d′

The sensitivity index *d′* is a crucial metric in signal detection theory, evaluating the participant's sensitivity level. However, determining the expected sensitivity corresponding to 70% balanced accuracy presents a challenge, as this performance level can arise from multiple combinations of hit rates and false alarm rates. To characterize the range of possible sensitivity values consistent with our target accuracy, we systematically identified 13 representative hit rate and false alarm rate pairs at five-percentage-point intervals spanning a plausible range (i.e., HR = 0.4, FAR = 0; HR = 0.45, FAR = 0.05; HR = 0.5, FAR = 0.1; HR = 0.55, FAR = 0.15; HR = 0.6, FAR = 0.2; HR = 0.65, FAR = 0.25; HR = 0.7, FAR = 0.3; HR = 0.75, FAR = 0.35; HR = 0.8, FAR = 0.4; HR = 0.85, FAR = 0.45; HR = 0.9, FAR = 0.5; HR = 0.95, FAR = 0.55; HR = 1, FAR = 0.6). These pairs ranged from extremely conservative responding (HR = 0.40, FAR essentially zero) through balanced moderate responding to extremely liberal responding (HR essentially 1.0, FAR = 0.60), all satisfying the 70% accuracy constraint. In the simulated data, a small constant was added to HR and FAR values in order to prevent infinite values arising from extreme proportions in calculating *d′* and criterion (Macmillan & Creelman, 2004). To estimate expected sensitivity and criterion without bias toward any single arbitrary combination, we employed a hierarchical permutation procedure. At the first level, we randomly sampled one of the 13 pairs with equal probability, computed its *d′* or criterion value, and repeated this sampling 1,000 times before averaging across samples to produce a permutation-specific mean. At the second level, we repeated this entire first-level procedure 1,000 times, yielding 1,000 permutation-specific means, which we then averaged to obtain stable final estimates. The hierarchical structure ensures that the final estimates reflect the central tendency across repeated random sampling from the feasible space rather than being determined by arbitrary selection of a single combination or being distorted by extreme outliers.

#### Computational validation of the robustness of a bias-free staircase method against interindividual differences in decisional bias

To directly test whether threshold differences across methods reflect criterion contamination versus other factors, we conducted a computational simulation isolating criterion effects while holding sensitivity constant (see Supplementary Materials). Virtual observers with varying decision criteria (conservative, neutral, liberal) but identical balanced accuracy completed threshold estimation using all four methods, enabling quantification of criterion-induced biases.

#### Test–retest reliability of the bias-free staircase method

To assess the temporal stability of the bias-free staircase method, we conducted a test–retest reliability analysis in an independent sample of participants. A total of 25 participants completed the identical bias-free staircase procedure on two separate occasions (T1 and T2). The same experimental parameters, stimulus characteristics, and adaptive rules were used in both sessions. Reliability was assessed at both the group and individual levels. At the group level, we compared mean threshold estimates and their associated standard errors across sessions. At the individual level, we evaluated relative reliability using intraclass correlation coefficients (ICC) derived from a two-way mixed-effects model with single measurements. Both absolute agreement [ICC(A,1)] and consistency [ICC(C,1)] were computed. In addition, Pearson correlation coefficients were calculated to provide a complementary measure of association between sessions. To characterize uncertainty around the reliability estimates, percentile bootstrap confidence intervals were obtained using 5,000 subject-level resamples. Additionally, we verified convergence on target performance by testing whether accuracy in test blocks using the estimated thresholds matched the intended 70% level. Full details of the reliability analysis are reported in the Supplementary Materials.

### Statistical analysis

First, we evaluated whether the contrast values extracted by means of the four methods for standard threshold were different. To this end, we ran a within-subject analysis of variance (ANOVA) having as a factor the four methods and as dependent variables the estimated thresholds. Subsequently, we calculated the accuracy, *d′*, and criterion based on the four test blocks to test for any differences between the methods in achieving the targeted level of accuracy (70%). To this end, we conducted an ANOVA having as a factor the four different methods and as dependent variables the accuracy, *d′*, and criterion. For tests where no significant differences were found, we computed Bayes factors (BF) to quantify evidence for the null hypothesis. BF > 3 provides substantial evidence against the null hypothesis, while BF < 1/3 provides evidence against the alternative hypothesis. In addition, mathematical simulations have revealed that a perceptual accuracy level of 70% is matched by a *d′* of approximately 1.46. Therefore, we also assessed how the calculated *d′* in the four test blocks aligned with this target value.

## Results

### Bias-free staircase yields systematically lower perceptual thresholds

We conducted an ANOVA to evaluate the presence of significant effects on the extracted threshold procedures resulting from the inclusion of catch trials in both constant stimuli and staircase methods. The analysis revealed a significant difference between the methods employed, *F*(3, 204) = 16.47, *p* < 0.001. Post hoc analysis (*p*-value corrected = 0.008, 6 comparisons) revealed that the contrast value extracted using the bias-free staircase method (Threshold_bias-free staircase_ = 33/222 ± 1.91 RGB) was significantly lower than the other extracted threshold values, all *t*(68) < −3.47, all *p* < 0.008. Moreover, the classic staircase method resulted in higher mean threshold values (Threshold_classic staircase_ = 44/211 ± 2.35 RGB) than both constant stimuli methods. However, the paired *t*-test did not reach the corrected level of significance, all *t*(68) > 2.37; all *p* > 0.009. Critically, Bayesian analysis revealed evidence for differences between classic staircase and constant stimuli methods (BF Threshold_classic staircase_ vs. Threshold_constant stimuli_ = 3.24; BF Threshold_classic staircase_ vs. Threshold_bias-free constant stimuli_ = 2.21), indicating that the nonsignificant corrected *p*-values may reflect overly conservative multiple-comparison correction rather than absence of true differences. Specifically, the comparison with classic constant stimuli showed substantial evidence for a true difference (BF = 3.24), while the comparison with bias-free constant stimuli showed anecdotal evidence (BF = 2.21). This pattern reveals a systematic gradient in threshold estimates: bias-free staircase yielded the lowest thresholds, classic staircase the highest, with constant stimuli methods intermediate. In contrast, the two constant stimuli methods did not exhibit different threshold estimation levels [Threshold_bias-free constant stimuli_ = 39/216 ± 1.64 RGB, Threshold_constant stimuli_ = 40/215 ± 1.88 RGB, *t*(68) = 1.2, *p* = 0.23, BF = 0.26]. Computational simulation directly validated this gradient as reflecting progressive criterion contamination (Supplementary Materials). Virtual observers with conservative criteria (matching our sample's mean *c*, see below) produced threshold estimates following the identical rank order: bias-free staircase converged correctly at the target intensity (mean bias = +0.16 RGB), while classical methods yielded progressively inflated estimates. As can be observed from Fig. 3, in both staircase methods, proper convergence of the value was demonstrated as the trials progressed. Additionally, in both constant stimulus methods, participants' average responses strictly followed a sigmoidal distribution (Fig. 2).

#### Only the bias-free staircase achieves target performance in an independent test

**Fig. 4:**
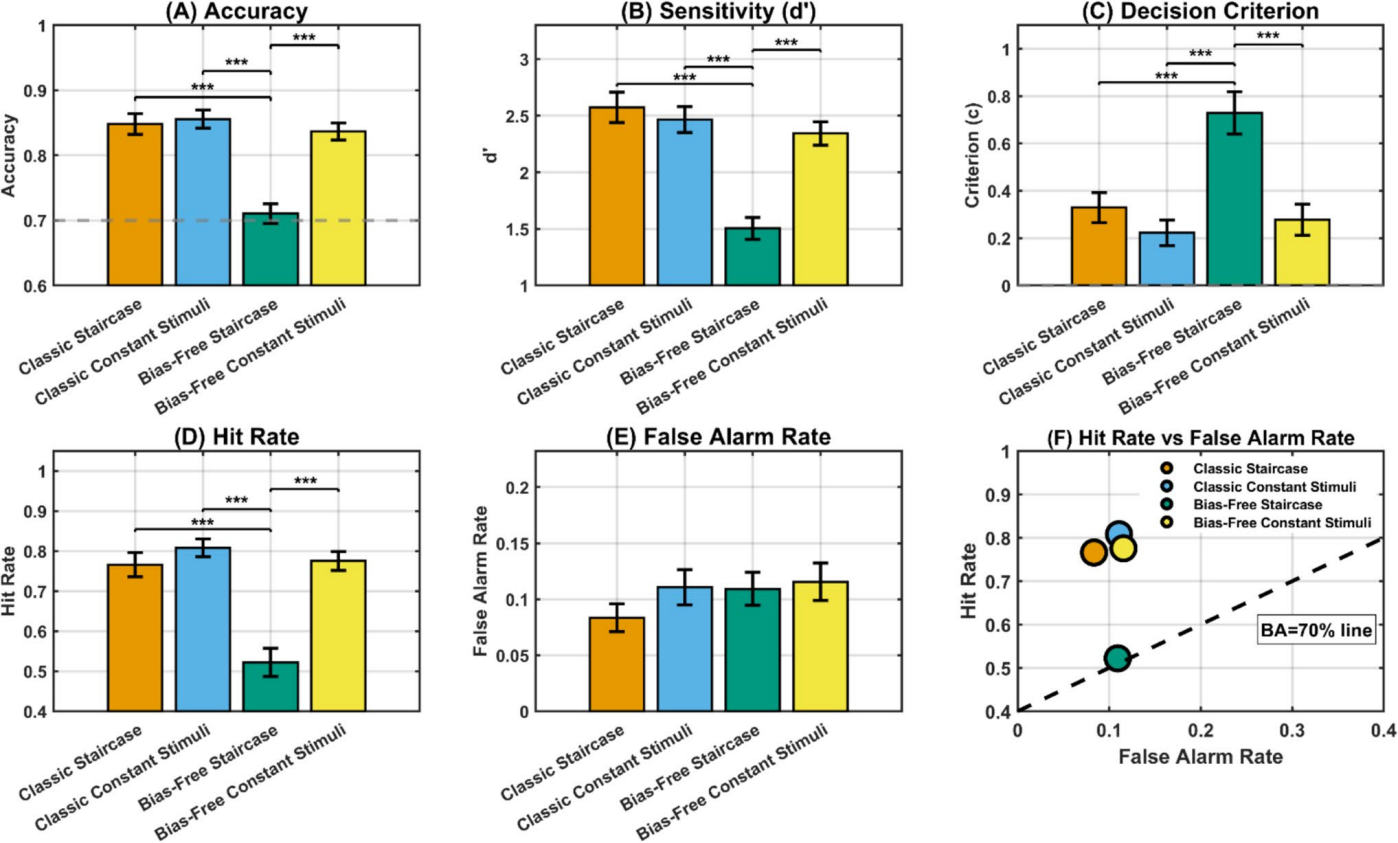
Comparison of psychophysical performance across four threshold estimation methods. Sixty-nine participants completed detection test blocks at thresholds estimated by four methods: classic 2-down/1-up staircase (orange), classic constant stimuli with psychometric fitting (blue), bias-free staircase (green), and bias-free constant stimuli with psychometric fitting (yellow). Bars represent group means, error bars indicate standard error of the mean (SEM), and significance markers denote pairwise comparisons (*p < 0.05, **p < 0.01, ***p < 0.001), which were reported only when the corresponding omnibus ANOVA was significant. **A** Test block accuracy. Bias-free staircase achieved target performance, while all classical methods significantly overestimated accuracy (all *p* < 0.001 vs. bias-free). Classical methods did not differ from each other (all *p* > 0.22). **B** Sensitivity (*d'*). Bias-free staircase yielded significantly lower *d'* estimates (1.50) than all classical methods (2.34–2.57, all *p* < 0.001), which did not differ among themselves (all *p* > 0.13). The bias-free estimate aligns with the theoretical expectation of 70% accuracy (*d'* = 1.46), while classical methods produced inflated values. **C** Decision criterion (*c*). Participants adopted significantly more conservative criteria (higher *c* values) in bias-free staircase (*c* = 0.73) than in the other methods (*c* = 0.22–0.33, all *p* < 0.001), which did not differ from each other (all *p* > 0.11). Dashed line indicates neutral criterion (*c* = 0). **D** Hit rate. Classical methods produced systematically higher hit rates (77–81%) than bias-free staircase (52%, all *p* < 0.001) but did not differ from each other (all *p* > 0.15), indicating that both the classical thresholds and bias-free constant stimuli approach present above-threshold stimuli that are easier to detect. **E** False alarm rate. False alarm rates remained relatively stable across methods, *F*(3, 204) = 2.52, *p* = 0.059; range, 8–12%. **F** Black dashed line represents the 70% balanced accuracy (BA) constraint (HR = 0.40 + FAR). Bias-free staircase converges near this target line (mean HR = 52%, FAR = 11%), while classical methods cluster above it (mean HR 77–81%, FAR 8–12%), indicating systematic threshold overestimation primarily through inflated hit rates rather than altered false alarm patterns.

We next evaluated threshold validity by testing all participants at each estimated threshold in an independent test block. This Stage 2 assessment examined which threshold estimation procedure (Stage 1) produced thresholds yielding performance closest to the target 70% accuracy level. A significant difference emerged between the bias-free staircase method and the other three methods, *F*(3, 204) = 26.05, *p* < 0.001 (Fig. 4A), when considering the accuracy achieved by participants. Specifically, the accuracy participants reached using the contrast value estimated with the bias-free staircase method was 71% ± 1%, far below the accuracy reached with the classic staircase method [accuracy = 84% ± 1%, *t*(68) = −6.24, *p* < 0.008], the classic constant stimuli [accuracy = 85% ± 2%, *t*(68) = −8.10, *p* < 0.008], and the bias-free constant stimuli [accuracy = 84% ± 1%, *t*(68) = −5.80, *p* < 0.008]. Crucially, no difference emerged when the other methods were compared, all *t*(68) < 1.22, all *p* > 0.22, all BF < 0.27. The results showed that three out of four methods overestimate the value of the perceptual threshold, underestimating participants' abilities. This, in turn, led to excessively high accuracy percentages in the test block. In contrast, the threshold extracted from the bias-free staircase method was very close to the target level of accuracy. To corroborate this result, we employed a one-sample *t*-test to assess whether the accuracy value extracted with the bias-free staircase method deviated significantly from the target value of 70%. The analyses indicated no significant difference, *t*(68) = 0.69, *p* = 0.49, BF = 0.17, suggesting a consistent alignment between the target accuracy and the accuracy observed in the bias-free staircase test block. Crucially, when repeating the same analysis on the accuracy levels obtained from the other three methods, all exhibited a significant deviation from the targeted accuracy, all *t*(68) > 9.19, all *p* < 0.001. Crucially, in an independent sample (*N* = 46), we validated the generalizability of the bias-free staircase method by conducting a supplementary study targeting 90% accuracy, confirming that the bias-free staircase successfully converges on target performance across difficulty levels (see Supplementary Materials).

Moreover, to ensure that the differences between the bias-free staircase and classic staircase did not derive from the blocked structure and/or accuracy-based adaptation, we conducted control experiments in an independent sample (*N* = 25) with a blocked version of the classic staircase. This control used an identical block structure (10 target-present trials per block) and identical accuracy-based adjustment rules, but calculated accuracy as hit rate only (without catch trials to measure false alarms). The blocked classic staircase produced thresholds (39.8 ± 2.8 RGB, mean ± SEM) statistically indistinguishable from the standard trial-by-trial classic staircase [44.2 ± 2.4 RGB; *t*(92) = 0.95, *p* = 0.34, BF = 0.36], confirming that the blocked presentation format per se does not alter threshold estimates. Critically, test block accuracy using the blocked classic staircase threshold was 79.5% ± 3.1%, significantly exceeding the 70% target, *t*(24) = 3.11, *p* = 0.005, mirroring the overestimation observed with the standard classic staircase. This confirms that blocked presentation and accuracy-based adjustment rules alone are insufficient to achieve target performance; rather, the inclusion of catch trials in the accuracy calculation, enabling explicit correction for response bias through the balanced accuracy formula, is the critical factor enabling bias-free threshold estimation.

Another investigation conducted to assess the stability of these methods focused on quantifying the percentage of thresholds that resulted in accuracy in the test block significantly above (i.e., ceiling, ≥ 90%) or below (i.e., chance, 50%) the target value of 70% accuracy. Crucially, while the bias-free staircase method yielded a significant deviation from the target value in 14% of results (7.2% ceiling, 7.2% chance), the other methods produced a substantial number of outcomes far from the target [classic staircase = 49% (49.3% ≥ 90%, 0.0% chance), classic constant stimuli = 51% (47.8% ceiling, 2.9% chance), bias-free constant stimuli = 33% (31.9% ceiling, 1.4% chance)]. This pattern of failures is highly informative. Classical methods fail almost exclusively by overestimating thresholds (ceiling effects, 47.8–49.3%), with essentially no underestimations (0–2.9% below chance). This asymmetry suggests that classical methods consistently produce thresholds that are too high, making test stimuli too easy. In contrast, the bias-free staircase shows a more balanced failure pattern (7.2% ceiling, 7.2% chance), with notably more chance-level performance than other methods. The fact that the bias-free approach produces both ceiling and floor errors (rather than only ceiling errors) indicates that it surrounds the target with errors in both directions, consistent with unbiased estimation. However, the total failure rate remains dramatically lower (14.5% vs. 33–51%), demonstrating superior overall stability despite this bidirectional variability.

#### Sensitivity analysis confirms proper calibration of the bias-free staircase

The same pattern of results emerged when we analyzed the sensitivity index (*d′*), *F*(3, 204) = 26.14, *p* < 0.001 (Fig. 4B). Specifically, *d′* was lower using the contrast value estimated with the bias-free staircase method than that obtained with the other conditions [*d′*_bias-free staircase_ = 1.50 ± 0.01, all *t*(68) < −5.8, all *p* < 0.008], which did not show any difference between each other [all *t*(68) < 1.50, all *p* > 0.13, *d′*_classic staircase_ = 2.57 ± 0.01, *d′*_constant stimuli_ = 2.47 ± 0.01, *d′*_bias-free constant stimuli_ = 2.34 ± 0.01, all BF < 0.41]. A *d′* value that is too high (above 2 and approaching 3), as found in three out of four methods, indicates excessively high performance compared to the desired level, while a *d′* of 1.50 emerging using the bias-free staircase method threshold is in line with the level of sensitivity we targeted.

To corroborate this result, we employed a one-sample *t*-test to assess whether the *d′* index extracted with the bias-free staircase method deviated significantly from the target value of *d′* that would be expected given 70% accuracy. Computational analysis demonstrated expected sensitivity of 1.46 (95% CI 1.42–1.50, see Supplementary Materials). The theoretical and observed values did not differ significantly [difference of 0.04 *d′* units, *t*(68) = 0.46, *p* = 0.64], with BF of 0.15 providing strong evidence for equivalence, confirming that participants' performance aligned with theoretical expectations for the target accuracy level. Crucially, when the same analysis was repeated for the sensitivity levels obtained from the other three methods, all exhibited a significant deviation from the targeted accuracy, all *t*(68) > 8.20, all *p* < 0.001. Further evidence of the robustness of the bias-free staircase emerges from direct analysis of the threshold estimation phase (Table 1), which provides prospective validation of our mechanistic account. Critically, method differences manifest during titration itself, not merely in subsequent testing. For the two bias-free methods incorporating catch trials, we could assess whether successful convergence on target performance occurred during titration. The bias-free staircase achieved performance closely matching the theoretical target in the final blocks used for threshold calculation (*d′* = 1.54 ± 0.07, accuracy = 73.2% ± 1.3%). In contrast, bias-free constant stimuli exceeded target levels even during threshold estimation (*d′* = 2.06 ± 0.07; accuracy = 77.1% ± 1.1%). This demonstrates that adaptive, real-time correction for criterion effects enables accurate threshold convergence, whereas post hoc psychometric fitting cannot achieve equivalent calibration despite including catch trials. Hit rate patterns reveal the mechanistic basis of these differences. Classical methods showed extreme values (classic staircase, 69%; constant stimuli, 53%). The 2-down/1-up rule converges to ~71% by design (Levitt, 1971), concentrating trials near threshold, while constant stimuli samples seven fixed levels including many subthreshold and many suprathreshold trials, yielding a lower aggregate HR. The two bias-free methods showed intermediate hit rates (bias-free staircase, 57%; bias-free constant stimuli, 61%). However, hit rate alone does not predict success: the decisive factor is the HR–FAR balance. The bias-free staircase achieved balanced performance (HR = 57%, FAR = 13%), yielding target sensitivity (*d′* = 1.54). Conversely, bias-free constant stimuli showed imbalanced performance (HR = 61%, FAR = 7%), reflecting extreme conservatism (*c* = 0.71) and resulting in inflated sensitivity (*d′* = 2.06). Post hoc fitting cannot correct for criterion-induced imbalance during data collection, whereas the real-time accuracy calculation in the bias-free staircase approach continuously balances hits and false alarms, achieving target performance during titration itself. Analysis by *t*-test further supported the absence of a significant difference between the *d′* values obtained during threshold estimation and the test block in the bias-free staircase method, *t*(68) = 0.33, *p* = 0.74, BF = 0.13. However, this was not the case in the bias-free constant stimuli approach, where a significant difference emerged, *t*(68) = 2.23, *p* = 0.03. Similarly, when examining accuracy, the bias-free staircase method did not exhibit significant modulations, *t*(68) = 1.11, *p* = 0.27, BF = 0.23, in contrast to the bias-free constant stimuli approach, where a significant difference was evident, *t*(68) = 6.88, *p* < 0.001. It is worth noting that the same analyses could not be conducted on the two classical methods due to the absence of catch trials, which prevented the calculation of *d′* values.

**Table 1:** Performance metrics during the threshold estimation phase (Stage 1) for the different psychophysical methods

Method	Threshold (RGB)	Accuracy (%)	Hit rate (%)	FAR (%)	*d′*	Criterion
Classic staircase	43.8 ± 2.3	N/A	69 ± 2	N/A	N/A	N/A
Constant stimuli	40.0 ± 1.2	N/A	53 ± 2	N/A	N/A	N/A
Bias-free staircase	33.0 ± 1.9	73 ± 1	57 ± 3	13 ± 2	1.54 ± 0.07	0.54 ± 0.07
Bias-free constant stimuli	39.0 ± 0.2	77 ± 1	61 ± 2	7 ± 1	2.06 ± 0.07	0.71 ± 0.06

Reported values include threshold estimates (RGB units), accuracy, hit rate, false alarm rate (FAR), sensitivity (*d′*), and response criterion (*c*). Thresholds and performance measures are reported as mean ± SEM across participants. Signal detection theory metrics (*d′* and criterion) are shown only for bias-free methods, for which both hit and false alarm rates were available

#### Criterion and response decomposition reveal the mechanism of failure of classical methods

Furthermore, we found a significant modulation of the decision criterion based on the method used, *F*(3, 204) = 15.13, *p* < 0.001 (Fig. 4C). Specifically, criterion was higher in the test block employing the contrast value estimated with the bias-free staircase method compared to the others [*c*_bias-free staircase_ = 0.73 ± 0.09, all *t*(68) > 3.98, all *p* < 0.008], which did not show any difference between each other [all *t*(68) < 1.62, all *p* > 0.11, *c*_classic staircase_ = 0.33 ± 0.06, *c*_constant stimuli_ = 0.22 ± 0.05, *c*_bias-free constant stimuli_ = 0.28 ± 0.07, all BF < 0.46]. This result indicates that participants adopted a more conservative criterion in the bias-free staircase test block compared to the other methods, which exhibited a less pronounced conservative tendency. However, a conservative criterion was generally adopted across the four methods (a one-sample *t*-test against 0 revealed a significant deviation of the criterion from 0, all *t*(68) > 4.13, all *p* < 0.001.

Critically, these positive criterion values reflect different underlying mechanisms depending on task difficulty. In the bias-free staircase condition, where stimuli were presented at genuinely threshold-level intensities (*d′* = 1.50, accuracy = 70%), the strongly conservative criterion (*c* = 0.73) represents an adaptive response to high perceptual uncertainty. Under signal detection theory, when signal detection is difficult (narrow separation between signal and noise distributions), observers face greater uncertainty and tend to adopt conservative criteria to minimize costly false alarms (Rahnev & Denison, 2018). In contrast, in classical methods, signal and noise distribution become widely separated, and criterion placement becomes less consequential for performance outcomes. Under such high-sensitivity conditions, SDT criterion estimates become “coarsened,” i.e., small variations in criterion produce minimal changes, making the estimated criterion parameter insensitive to the observer's actual decision policy. Thus, while all methods yielded positive criterion values, only the bias-free staircase's estimate reflects a genuine, interpretable conservative shift; the others likely reflect ceiling effects that mask criterion variability. Thus, an estimated threshold exceeding the observer's actual capabilities does not merely inflate sensitivity levels in the test block to excessive degrees; paradoxically, it also coarsens criterion estimates. This occurs because overly high sensitivity masks potential response trends, rendering the criterion measure insensitive. To provide a complete characterization of response patterns and to avoid potential limitations of equal-variance SDT assumptions, we also analyzed hit rates (HR) and false alarm rates (FAR) separately (Fig. 4D, E). Hit rates differed substantially across methods: classic staircase (*M* = 77% ± 3% SEM), classic constant stimuli (*M* = 81% ± 2%), bias-free staircase (*M* = 52% ± 4%), and bias-free constant stimuli (*M* = 78% ± 2%). A repeated-measures ANOVA confirmed a significant effect of method, *F*(3, 204) = 23.68, *p* < 0.001. Post hoc paired *t*-tests revealed that the bias-free staircase yielded significantly lower hit rates than all other methods (all *p* < 0.001), while the other three methods did not differ significantly from one another (all *p* > 0.15, all BF < 0.35). This pattern indicates that bias-free threshold estimation presents genuinely threshold-level stimuli during testing, whereas classical methods present above-threshold stimuli that are easier to detect. False alarm rates showed greater stability across methods: classic staircase (*M* = 8% ± 1%), classic constant stimuli (*M* = 11% ± 2%), bias-free staircase (*M* = 11% ± 2%), and bias-free constant stimuli (*M* = 12% ± 2%). The effect of method was only marginal, *F*(3, 204) = 2.52, *p* = 0.059. Post hoc tests revealed modest differences between the classic staircase and constant stimuli approaches (classic staircase vs. classic constant stimuli, *t* = −2.34, *p* = 0.022, BF = 1.68; classic staircase vs. bias-free constant stimuli, *t* = −2.60, *p* = 0.011, BF = 2.96), but no significant differences between bias-free staircase and other methods (all *p* > 0.05, all BF < 0.75). This asymmetry—i.e., strong effects on hit rates but minimal effects on false alarm rates—provides critical insight into how classical methods fail. The bias manifests primarily as inflated hit rates during testing (reflecting above-threshold stimulus presentation) rather than as altered false alarm patterns (Fig. 4F). Notably, the bias-free staircase hit rate is dramatically lower than the other methods, representing an approximately 30% reduction. In contrast, false alarm rates remain comparable across methods (all within 8–12%), with the bias-free method (11%) falling in the middle of this narrow range. This decomposition validates our SDT-based interpretation while demonstrating that the overestimation problem in classical methods is fundamentally about stimulus strength (HR differences) rather than criterion contamination affecting FA rates during testing.

#### Bias-free staircase yields stable and reliable threshold estimates

The bias-free staircase method showed good temporal stability across testing sessions (see Supplementary Materials). Group-level threshold estimates were highly consistent between sessions (T1 = 36.08 RGB; T2 = 36.32 RGB), with nearly identical standard errors (SEM T1 = 2.59 RGB; SEM T2 = 2.63 RGB), indicating stable estimation at the population level. At the individual level, threshold estimates were strongly correlated across sessions (*r* = 0.78, *p* < 0.001). Intraclass correlation analyses revealed good reliability, with an ICC(A,1) of 0.79 and an ICC(C,1) of 0.78. Bootstrap-derived 95% confidence intervals indicated that reliability was consistently above moderate levels. No systematic shift between test and retest was observed, as the mean within-subject difference (T2 − T1) was close to zero [0.24 RGB; *t*(24) = 0.14, *p* = 0.89]. Critically, thresholds identified by the bias-free staircase yielded target performance in this independent sample, with test block accuracy averaging 71.6% ± 2.6%, not differing significantly from the 70% target, *t*(24) = 0.62, *p* = 0.54, BF = 0.25. Together, these results demonstrate that the bias-free staircase method yields stable threshold estimates across sessions, supporting its reliability as a psychophysical tool.

## Discussion

Estimating perceptual thresholds involves determining the minimum intensity level of a stimulus leading an individual's perceptual system to accurately detect a sensory input. This process is crucial in various fields such as psychology and neuroscience in which the perceptual threshold value is correlated with cognitive (Bidelman et al., 2013; Calvo & Esteves, 2005; Sara & Faubert, 2000) and physiological indices (Newsome et al., 1989; Romei & Tarasi, 2026; Tarasi & Romei, 2023; Tucker et al., 1989; van Stiphout et al., 2022). This initial calibration of the stimulus intensity forms a crucial preparatory step of many studies, ensuring that stimuli are presented at appropriate levels to elicit desired responses from participants (Cochrane et al., 2023; Steenbergen et al., 2012; Tarasi, Alamia et al., 2026; Tarasi, di Pellegrino, et al., 2022; Watamaniuk et al., 2003).

However, current methods for estimating perceptual thresholds have a fundamental issue. They fail to account for decisional bias during threshold estimation, resulting in an inaccurate estimate, casting doubt on the reliability of the results built upon the measured threshold. Given that threshold estimation forms the basis of numerous psychophysical studies, this issue is particularly concerning. A clear example appears in Gallina et al. (2024), who used a Bayesian adaptive procedure to estimate detection thresholds for each participant without measuring false alarm rates. Participants were then tested at their individually estimated thresholds in blocks containing catch trials, enabling the calculation of *d′* from test block performance. Based on their performance, participants were categorized as “good performers” or “bad performers.” However, this approach is critically compromised by criterion contamination during threshold estimation, as the titration phase converges based solely on signal-present trial responses, producing threshold estimates that confound sensitivity with criterion. As our data demonstrate, classical thresholds are strongly associated with criterion while bias-free estimates are not. Consequently, conservative responders yield inflated thresholds, causing test stimuli to be presented well above the true threshold, artificially inflating test block *d′* and producing “good performer” classification despite potentially average sensitivity. Conversely, liberal responders yield deflated threshold estimates, resulting in test stimuli being presented below the true perceptual threshold. This, in turn, artificially reduces *d′* in the test block, increasing the risk of misclassifying such participants as “bad performers.” Moreover, several (sub)clinical conditions exhibit inherent biases in decision-making. For instance, individuals on the autism spectrum lean toward a conservative approach, gathering extensive bottom-up evidence before reaching a decision (Brosnan et al., 2014; Quinde-Zlibut et al., 2020; Tarasi et al., 2021; Tarasi, Trajkovic, et al., 2022a, 2022b; Tarasi, Borgomaneri, et al., 2023; Tarasi et al., 2024; Ursino et al., 2022). Conversely, those within the schizophrenic spectrum tend to adopt a more liberal criterion (Parola et al., 2021; Tarasi, Martelli, et al., 2023a, 2023b). Therefore, not controlling for decisional bias allows these traits/conditions to influence the threshold estimate due to their tendency to rigidly adopt a specific criterion.

To address this issue, we proposed integrating trials without target (*catch trials*) during threshold estimation, enabling a more precise measurement by accounting for both FAR and HR. To test this approach, we compared the most widely used approaches for thresholds determination—staircase and constant stimuli methods—with our own modified methods, taking into account and controlling for the decisional bias. Subsequently, we assessed the precision and reliability of the estimated thresholds by considering accuracy and sensitivity (*d′*) in a test block.

Results showed a significant difference between the bias-free staircase and the other three methods. While the bias-free staircase method achieved the targeted accuracy of 70% in the test block, the other three significantly exceeded this threshold (i.e., 85%), suggesting a tendency to overestimate participants' perceptual thresholds. Crucially, we also validated the generalizability of the bias-free staircase method by targeting 90% accuracy—beyond the 70% accuracy level—in an independent sample (see Supplementary Materials). The bias-free staircase successfully achieved this substantially higher target (90.4% ± 1.0%). Moreover, by analyzing the performance directly in the titration phase, we showed that the bias-free staircase (but not the bias-free constant stimuli) achieved target performance (accuracy = 73%) during threshold estimation itself, demonstrating that real-time accuracy-based adaptation successfully eliminates criterion contamination as thresholds converge. Importantly, the bias-free staircase also showed good temporal stability, with test–retest analyses revealing reliable threshold estimates across sessions that consistently converged on target performance (see Supplementary Materials).

In the test block, the bias-free staircase approach also led to a different level of sensitivity (*d′*) compared to other methods. Bias-free staircase yielded notably lower *d′* values (*d′* = 1.5) than the others, which had very high values (*d′* = 3), meaning that participants too easily discriminated between trials with and without the target (i.e., close to ceiling performance), rarely making errors. These significant differences in both accuracy and *d′* observed in the bias-free staircase suggest that the other procedures were excessively easy for participants. The excessive ease found in the classic staircase approach and constant stimuli methods has cascading consequences for interpreting test block performance. According to SDT, the simpler the discrimination (high *d′*), the less impactful the placement of the decision criterion will be in determining decision outcomes (Green & Swets, 1966). Therefore, in classical methods, a paradoxical scenario unfolds wherein the decision criterion exerts a strong influence in modulating the resultant threshold, with liberal criteria linked to a lower threshold compared to a conservative one. However, in the test block, when these overestimated thresholds present above-threshold stimuli, behavioral differences become characterized by ceiling-level performance. Our decomposition of test block performance into hit rates and false alarm rates directly validates this interpretation. While classical methods showed dramatically inflated hit rates (77–81%) compared to the bias-free method (52%), reflecting the presentation of above-threshold stimuli, false alarm rates remained remarkably stable across all methods (8–12%). This asymmetry demonstrates that the issue with classical methods manifests primarily as differences in stimulus detectability (large HR effects) rather than as ongoing differences in response bias during testing (minimal FAR effects). The stability of false alarm rates indicates that participants adopt broadly similar decision policies when responding to catch trials; the large differences in hit rates arise because classical methods present stimuli that are objectively easier to detect. Therefore, imprecise methods of threshold estimation render both sensitivity and criterion measures insensitive and inaccurate due to ceiling-level performance masking true perceptual parameters.

Crucially, of the two bias-free methods, only the bias-free staircase led to an improvement in threshold estimation, whereas the bias-free constant stimuli method did not prove equally effective. A possible interpretation is that the decision bias was only partially controlled. This reveals that merely adding catch trials is insufficient; rather, the critical factor is how false alarm information is integrated into threshold estimation. In our bias-free constant stimuli implementation, catch trials (0% contrast) constituted one of seven intensity levels presented to participants. The psychometric fitting procedure treated this as simply the lowest point on the curve, with the threshold extracted from a sigmoid fitted across all seven data points. This created an asymmetry wherein 15% of information (catch trials) constrained false alarm rates while 85% (six target-present levels) provided criterion-contaminated hit rates. Crucially, psychometric fitting is a post hoc curve-fitting procedure with no mechanism to correct hit rates based on observed false alarms; it simply finds the best sigmoid through all points, including contaminated ones. Even increasing the proportion of catch trials did not resolve this fundamental limitation (see Supplementary Materials), as the problem is architectural rather than quantitative.

An intriguing question further arises: why have imprecise methods led to an overestimation rather than an underestimation of the perceptual threshold? Once again, one potential explanation points to the uncontrolled role of the criterion. Upon analyzing the criterion in the test block, we showed that participants adopted a conservative bias. This finding mirrors other studies showing a tendency to favor a conservative versus liberal criterion in perceptual tasks (Rahnev & Denison, 2018). Crucially, we can trace how this conservative tendency would have produced overestimation in classical methods but not in bias-free methods. During threshold estimation in classical methods (which lack catch trials), a conservative criterion reduces hit rates without any counterbalancing information from false alarms. The staircase or fitting algorithm interprets these reduced hit rates as evidence of poor sensitivity and increases stimulus intensity accordingly, producing an overestimated threshold. In the bias-free staircase, the same conservative tendency reduces hit rates, but this reduction is explicitly balanced against false alarm rates in the accuracy calculation. The hit rate/false alarm rate decomposition confirms this mechanism. In the test block, bias-free thresholds produced dramatically lower hit rates because they correctly estimated sensitivity and presented genuinely threshold-level stimuli. Critically, false alarm rates remained very low and comparable across methods, indicating that the conservative criterion is present in all conditions but only affects threshold estimation when not explicitly controlled through catch trials. Therefore, our findings suggest that classical methods allowed conservative criteria during titration to inflate thresholds, which then presented easy-to-detect stimuli during testing (evidenced by high HR). This interpretation receives support from computational simulation (Supplementary Materials), which demonstrated that criterion contamination produces precisely the threshold pattern observed. When virtual observers with conservative criteria (FAR = 5%, matching our participants' profile) completed threshold estimation, classical methods converged at inflated intensities (+3.5 to +7.1 RGB bias) while bias-free staircase converged correctly at the target (bias = +0.16 RGB). Critically, the simulation achieved this by holding sensitivity constant across criterion groups, confirming that threshold differences reflect criterion effects rather than genuine sensitivity differences.

As we concentrated solely on visual perceptual thresholds, future investigations should aim to broaden the scope of this method to encompass the measurement of other types of thresholds, such as those related to auditory and tactile perception or memory tasks, facilitating the application of our findings across diverse fields of study. Furthermore, our identified technique has proven functional and holds significant potential to enhance the robustness of future studies focusing on visual detection tasks. However, it is worth noting that in discrimination tasks, such as the random dot motion task (Di Luzio et al., 2022), the influence of decisional bias tends to be less pronounced, since both decision scenarios (i.e., leftward vs. rightward movement) are present. Lastly, while we opted for the most representative methods in our study (i.e., staircase and constant stimuli methods), it is important for future investigations to consider comparing them with other traditional methods, most notably two-interval forced-choice (2IFC) (Iemi & Busch, 2018), which eliminate response bias by design. However, 2IFC measures discrimination (e.g., which interval differs) rather than absolute detection (is a signal present), engaging different cognitive operations and precluding measurement of decision criterion. Our bias-free yes/no detection method offers complementary advantages: it preserves construct validity for detection tasks, enables separate quantification of sensitivity and criterion (theoretically meaningful for studying decision bias), provides cleaner single-event neural signals for neuroimaging analyses, reduces cognitive load by eliminating interval comparison demands, and avoids temporal interference effects (masking, adaptation). Future investigations should also consider comparisons with other traditional methods such as JND (just noticeable difference; Stevens et al., 1975), as well as modern adaptive methods (e.g., Bayesian and maximum-likelihood methods like QUEST and PEST; Taylor & Creelman, 1967; Watson & Pelli, 1983). Such comparisons would provide additional evidence to support the effectiveness of the chosen approach and clarify its advantages relative to the broader landscape of psychophysical methods. Furthermore, the successful validation across difficulty levels (70% and 90%) has important practical implications. Researchers can flexibly select target accuracy based on experimental goals: lower targets (60–70%) for challenging detection tasks where sensitivity is the primary variable of interest, versus higher targets (80–90%) for experiments requiring minimal error.

In conclusion, the newly developed bias-free staircase approach allowed for more precise measurement of perceptual thresholds. Unlike other threshold estimation approaches, this method ensures that decisional criterion has less impact in dictating the resultant threshold. Compared to the classical methods, individuals with liberal tendencies are not disadvantaged during the test phase due to underestimated thresholds, and conservative individuals do not gain an advantage from overestimated thresholds. Crucially, the bias-free method does not eliminate but rather accounts for the decisional bias, allowing it to be identified and studied in the test block. Thus, although individuals with liberal or conservative criteria may persist in adopting their tendencies, this was shown not to impact the resultant threshold. This work is part of a line of research aimed at improving the classical methods used to collect perceptual thresholds (Buss et al., 2001; Fioravanti et al., 2022; Grassi & Soranzo, 2009; Kaernbach, 2001; Prins & Kingdom, 2018; Shen et al., 2015; van den Berg et al., 2022). However, it is the first to specifically propose a method taking decisional bias into account when estimating the threshold. Additionally, the applicability of this method is extremely broad, potentially fitting into a plethora of experimental paradigms from visual to auditory, tactile, and even memory tasks. Furthermore, the proposed procedure is simple and quick to execute, making it highly feasible to use, as its implementation simply requires creating mini-blocks where, in addition to target-present trials, an equal number of catch trials are presented. The method will adapt to the participant's actual sensitivity based on their objective accuracy, discarding any criterion-related influence. Therefore, we advocate for the adoption of this straightforward, bias-free method to boost the reliability of threshold estimation in future scientific investigations.

## Supplementary Information

Below is the link to the electronic supplementary material.

Supplementary file1 (DOCX 615 KB)

## Data Availability

All study primary data are publicly available on figshare (10.6084/m9.figshare.28742918.v1).

## Code availability

Original devised code has been made publicly available on figshare (10.6084/m9.figshare.28742918.v1).
