# Supplementary material for: A novel bias-free approach for robust perceptual threshold estimation
Source: Behav Res Methods. 2026 May 5;58(6):157. doi: 10.3758/s13428-026-03038-5 (PMC13144207; doi:10.3758/s13428-026-03038-5)
Supplement: Supplementary file 1 — Supplementary file1 (DOCX 615 KB) [file 13428_2026_3038_MOESM1_ESM.docx]

**Validation at 90% Target Accuracy**

To assess whether the bias-free staircase's effectiveness generalizes beyond the 70% target used in the main study, we conducted a validation experiment targeting 90% accuracy in an independent sample. This higher threshold provides a stringent test of method sensitivity: if advantages are specific to 70%, performance should degrade at 90%; if the core mechanism is robust, the method should successfully converge on 90% during titration and maintain this in testing. Forty-six participants completed the identical detection task described in the main study, with the bias-free staircase threshold estimation procedure modified to target 90% rather than 70% accuracy. The accuracy-based adjustment rules were modified to converge on 90% rather than 70% (accuracy ≤ 0.70 → increase by 4 RGB; accuracy ≤ 0.80 → increase by 1 RGB; accuracy > 0.90 → decrease by 2 RGB). All other parameters (20 blocks, 5 catch + 5 target per block, threshold = mean of last 3 blocks) remained identical. Test Block Performance were the following:

- Accuracy: 90.4% ± 1.0%
- Sensitivity (d'): 3.20 ± 0.11
- Criterion: 0.075 ± 0.089
- Hit Rate: 88.3% ± 2.0%
- False Alarm Rate: 7.8% ± 1.2%

We demonstrated that accuracy was not different from the 90% target (t₄₅ = 0.40, p = 0.69) and that Criterion was not different from 0 (neutral) (t₄₅ = 0.84, p = 0.41). The high sensitivity (d' = 3.20) reflects the substantially easier detection task at this performance level compared to 70% target (d' = 1.5 in main study). This tight correspondence validates that the method achieves intended performance through genuine sensitivity calibration rather than artifact. Importantly, participants adopted near-neutral decision criteria (c = 0.075, not significantly different from 0) at 90% target, contrasting with the conservative criteria observed at 70% target. This suggests adaptive criterion adjustment based on task demands: at 70% (moderate difficulty with higher uncertainty), observers adopt conservative strategies to manage detection errors; at 90% (stimuli well above threshold with lower uncertainty), neutral criteria suffice. Critically, the method successfully calibrated performance regardless of criterion strategy demonstrating that accuracy-based adaptation corrects for criterion effects across the full range of criterion placements. The successful convergence at 90% is particularly noteworthy because this high accuracy level presents challenges for threshold estimation: fewer errors mean less information per trial, and any criterion bias has amplified effects when hit rates approach ceiling. Despite these challenges, the bias-free staircase maintained precise calibration (90.4% vs. 90.0% target), demonstrating robustness even under stringent conditions.

**Test–retest reliability (***bias-free staircase method***)**

To evaluate the stability of the bias-free staircase method across sessions, we assessed test–retest reliability in 25 participants who completed the same procedure twice (T1 and T2). At the group level, the average values were highly comparable across sessions (T1 mean = 36.08 RGB; T2 mean = 36.32 RGB). The standard errors of the mean were similarly small and nearly identical (SEM T1 = 2.59 RGB; SEM T2 = 2.63 RGB), indicating stable group estimates. We first examined whether there was any systematic shift between sessions by computing the within-subject difference (T2 − T1). The mean difference was close to zero (bias = 0.24 RGB), and a paired t-test indicated no evidence of a systematic change from test to retest (t_24_ = 0.14, p = 0.89). Relative reliability was quantified using an intraclass correlation coefficient (ICC) from a two-way mixed-effects model, single-measurement. Reliability was good (ICC(A,1) = 0.79). A very similar estimate was obtained for consistency (ICC(C,1) = 0.78). To characterize uncertainty, we derived percentile bootstrap confidence intervals using 5000 subject-level resamples. The resulting 95% confidence intervals were [0.44, 0.92] for both ICC(A,1) and for ICC(C,1). The width of these intervals indicates that, with N = 25, the ICC estimate is compatible with reliability ranging from moderate to very high. We also report the Pearson correlation between sessions, which was high (r = 0.78, p < 0.001). To verify that the bias-free staircase successfully converged on target performance in this independent sample, we assessed test block accuracy using the estimated thresholds (2e used, for each participant, the threshold obtained from the first staircase procedure). Mean accuracy was 71.6% ± 2.6% (SEM), closely matching the target of 70%. A one-sample t-test confirmed no significant deviation from target (t₂₄ = 0.62, p = 0.54, BF = 0.25), providing further evidence that the method reliably identifies intensities yielding criterion-free performance at the intended level across different samples and testing occasions.

**Increased catch trial proportion in constant stimuli did not improve threshold estimate.**

To empirically test whether increasing catch trial proportion resolves the bias-free constant stimuli method's limitations, we conducted a supplementary study (N=25) with catch trials increased from 100 to 120 (60% of trials). This 60:40 catch-to-target ratio approaches the practical maximum: further increases would leave insufficient trials per target level for reliable psychometric fitting. Threshold estimation and test procedures were otherwise identical to the main study. Despite 60% catch trials, the modified constant stimuli method still overestimated thresholds. Test block accuracy was 77.6% ± 2.9%, significantly exceeding the 70% target (t₂₄ = 2.62, p = 0.015). This overestimation confirms that increasing catch trial proportion, even beyond the 50% used in the successful bias-free staircase, cannot resolve the fundamental limitation of psychometric fitting. The architectural difference between adaptive adjustment (real-time criterion correction) and non-adaptive estimation (post-hoc curve fitting) is fundamental and cannot be overcome by optimizing trial composition alone.

**Computational model demonstrates that bias-free staircase is the only method resistant to criterion contamination in threshold estimation**

To directly test whether the bias-free staircase's advantages stem from its adaptive correction mechanism rather than merely including catch trials, we conducted a computational simulation isolating the effects of observer criterion on threshold estimation. We simulated virtual observers who differed exclusively in decision bias while maintaining identical balanced accuracy (70%) at a target intensity (RGB = 33). This design allowed us to assess whether threshold estimation methods correctly converge on the intensity where balanced accuracy equals the target, or whether criterion differences bias threshold estimates. The simulation modelled observers using a signal detection theory framework where hit rate depends on both detection sensitivity and decision criterion: HR(x) = FAR + (1-FAR)·g(x), where FAR quantifies criterion (false alarm rate on catch trials), and g(x) represents the "detection component" (i.e., a logistic function of stimulus intensity). Critically, we calibrated each criterion group's psychometric function such that all observers achieved balanced accuracy BA = (HR + [1-FAR])/2 = 0.70 at RGB = 33, despite having different false alarm rates. This calibration required adjusting the psychometric function's location parameter (θ) to compensate for criterion effects: observers with conservative criteria (low FAR) exhibited lower detection sensitivity g(x) at the target intensity, but this was offset by fewer false alarms, maintaining BA = 0.70. Conversely, liberal observers (high FAR) showed higher g(x) but more false alarms, again yielding BA = 0.70. This approach mirrors real-world scenarios where observers with different decision strategies may achieve equivalent balanced accuracy through different HR-FAR combinations. We simulated three criterion groups (N=100 per group): Conservative (FAR = 5%), Neutral (FAR = 20%), and Liberal (FAR = 35%). Each virtual observer completed threshold estimation using all four methods tested in the main study: (1) classic 2-down-1-up staircase, (2) bias-free staircase, (3) constant stimuli with psychometric fitting (no catch trials), and (4) constant stimuli with catch trials and psychometric fitting. All methods used identical parameters to the experimental procedures described in the main manuscript, including trial counts, step sizes, and convergence rules. Threshold estimation always began at RGB = 45, ensuring methods had to empirically search for the target performance level rather than being initialized near the true threshold. Repeated-measures ANOVA revealed significant main effects of both METHOD (F_3,297_ = 70.836, p < 0.001) and CRITERION (F_2,198_ = 778.595, p < 0.001), as well as a significant METHOD × CRITERION interaction (F_6,594_ = 97.034, p < 0.001). This interaction indicates that criterion contamination varied substantially across threshold estimation methods.


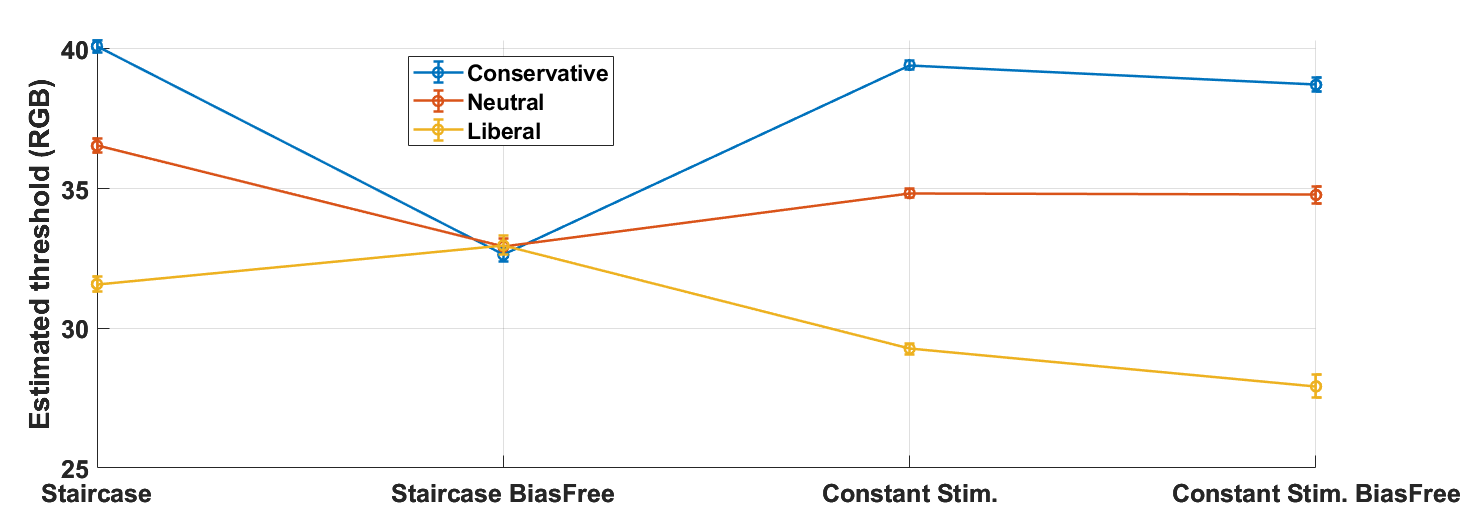


Method-specific analyses highlighted the nature of this effect. Classic 2-down-1-up staircase showed severe criterion contamination (F _2,198_ = 313.913, p < 0.001). Estimated thresholds varied systematically with observer criterion: Conservative observers yielded inflated thresholds (40.09 ± 2.25 RGB, +7.09 RGB bias), Neutral observers produced moderately elevated thresholds (36.53 ± 2.44 RGB, +3.53 RGB bias), and Liberal observers resulted in thresholds close to the true value (31.58 ± 2.78 RGB, -1.42 RGB bias). The between-group range was 8.51 RGB, indicating that criterion alone caused threshold estimates to vary by approximately 26% of the true threshold value. This pattern reflects the 2-down-1-up procedure's convergence to approximately 71% hit rate: conservative observers (with low baseline false alarm rates) required supra-threshold intensities to achieve 71% HR, while liberal observers (with high baseline FAR) easily reached 71% HR even at sub-threshold intensities. Bias-free staircase, in contrast, demonstrated robustness to criterion effects (F_2,198_ = 0.428, p = 0.653). Threshold estimates remained stable across criterion groups: Conservative 32.63 ± 2.51 RGB (-0.37 RGB bias), Neutral 32.92 ± 2.76 RGB (-0.08 RGB bias), and Liberal 32.97 ± 3.34 RGB (-0.03 RGB bias). The between-group range was 0.34 RGB, 25 times smaller than the classic staircase. All groups converged near the true threshold (overall mean = 32.84 RGB, SE = 0.17), with the minor deviation (0.16 RGB) falling well within measurement noise. The non-significant ANOVA confirms that observer criterion did not systematically influence threshold estimates, validating the method's core design principle: continuous correction for criterion effects through balanced accuracy calculation. Constant stimuli without catch trials (classic psychometric fitting) exhibited profound criterion contamination (F_2,198_ = 791.989, p < 0.001), producing the most extreme between-group differences. Conservative observers yielded substantially inflated thresholds (39.42 ± 1.68 RGB, +6.42 RGB bias), while Liberal observers produced severely deflated estimates (29.27 ± 1.97 RGB, -3.73 RGB bias), with Neutral observers intermediate (34.83 ± 1.67 RGB, +1.83 RGB bias). The between-group range reached 10.15 RGB, representing a 31% variation relative to the true threshold. Notably, the classic fitting method showed even greater criterion sensitivity than the classic staircase, likely because psychometric function fitting integrates responses across multiple intensity levels, amplifying the systematic bias introduced by criterion-contaminated hit rates. Constant stimuli with catch trials (bias-free fitting) also demonstrated significant criterion contamination (F _2,198_ = 273.570, p < 0.001), despite including 50% catch trials. Threshold estimates varied markedly: Conservative 38.71 ± 2.47 RGB (+5.71 RGB bias), Neutral 34.77 ± 3.06 RGB (+1.77 RGB bias), Liberal 27.92 ± 4.14 RGB (-5.08 RGB bias). The between-group range was 10.79 RGB, actually exceeding the classic fitting method without catch trials. This counterintuitive result, i.e., worse performance despite including catch trials, empirically confirms our theoretical prediction: merely adding catch trials to non-adaptive procedures is insufficient to eliminate criterion contamination.


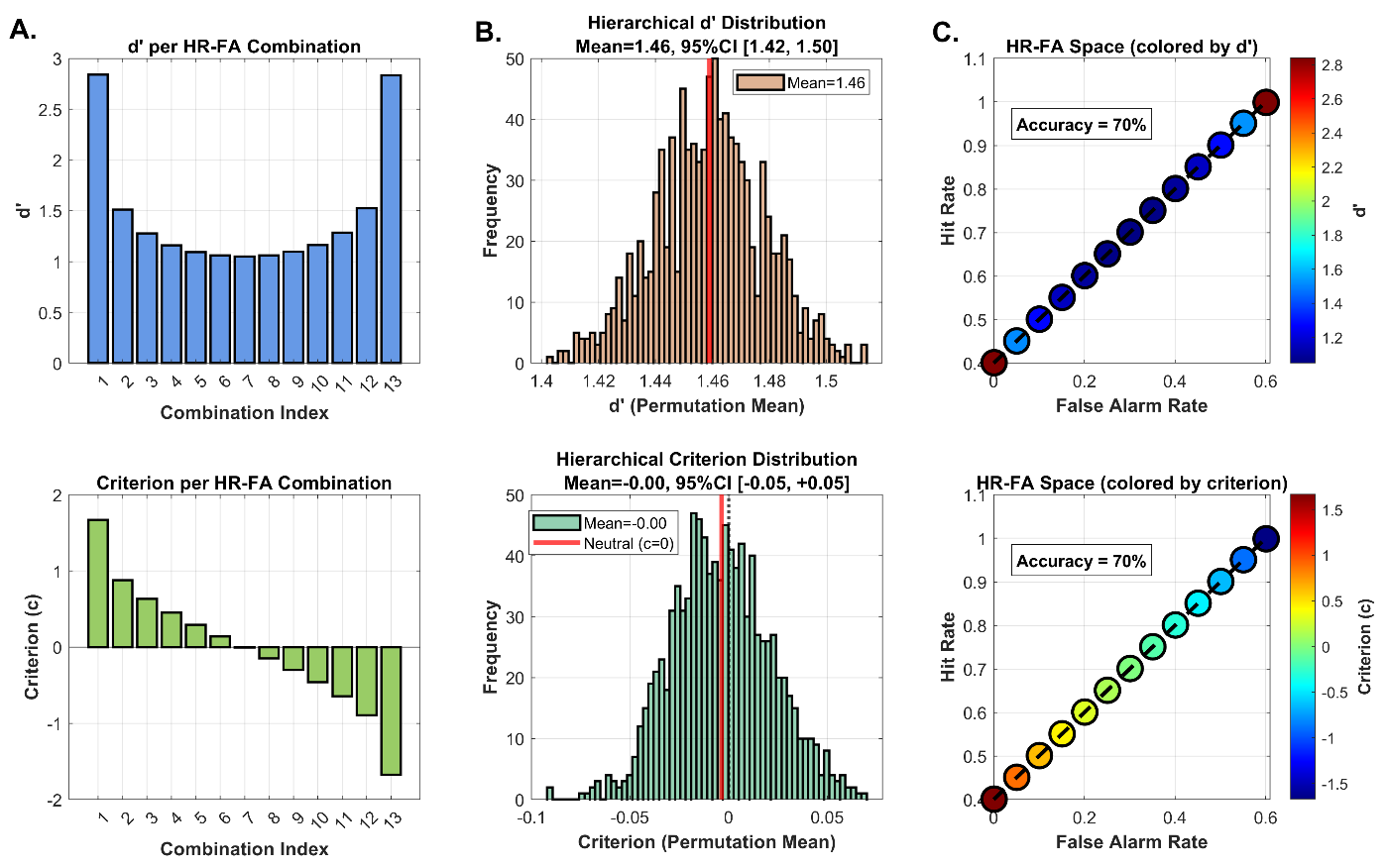


**Hierarchical permutation analysis of expected sensitivity and criterion for 70% balanced accuracy.**

(A) Sensitivity (d') and Criterion (c) values computed for each of the thirteen hit rate and false alarm rate combinations satisfying the 70% balanced accuracy constraint. Combinations are ordered from most conservative (index 1: HR=0.40, FAR≈0) to most liberal (index 13: HR≈1.0, FAR=0.60) criterion placement.

(B) Distribution of permutation-specific mean d' and criterion values obtained through hierarchical resampling (1000 iterations of 1000 samples each). Red solid line marks final estimate (d' = 1.46, 95% CI [1.42, 1.50]), demonstrating exceptional stability across random sampling procedures.

(C) Hit rate by false alarm rate space showing the thirteen combinations color-coded by sensitivity and criterion.
